# Psychometric properties and content of instruments for assessing penile cancer patients’ quality of life: A systematic review

**DOI:** 10.1016/j.ijnsa.2025.100412

**Published:** 2025-08-22

**Authors:** Anu Soikkeli-Jalonen, Suvi Vierelä, Antti Kaipia, Eeva Harju, Elina Haavisto

**Affiliations:** a Department of Health Sciences, Faculty of Social Sciences, Tampere University Arvo Ylpön katu 34, 33520 Tampere, Finland; b Department of Surgery, Tampere University Hospital, P.O. Box 272, 33101 Tampere, Finland; c Tampere University Hospital, Wellbeing Service County of Pirkanmaa, P.O. Box 272, 33101 Tampere, Finland

**Keywords:** Penile cancer, Quality of life, Instrument, Systematic review

## Abstract

**Aim:**

To describe the psychometric properties and content of the instruments used for assessing penile cancer patients’ quality of life

**Design:**

A systematic review

**Method:**

A systematic literature search was conducted in October 2024 across four electronic databases: PubMed, CINAHL, PsycINFO, and the Cochrane Library. The systematic approach was adhered to when conducting the review, and the Preferred Reporting Items for Systematic Reviews and Meta-Analyses guidelines were followed to ensure explicit reporting. The search across the four databases generated 135 articles, out of which 16 were included in the review.

**Results:**

Eight instruments assessing the quality of life (QoL) among penile cancer patients were identified: four generic, two cancer-specific, and two penile cancer-specific. The content of these instruments fell into three main categories: physical functioning, psychosocial resilience, and overall life functions. However, none of the instruments covered all these aspects comprehensively. Additionally, no instrument was reported to be thoroughly valid or reliable.

**Conclusions:**

A psychometrically tested and validated QoL instrument that covers all aspects of penile cancer patients' well-being was not found. There is a need for holistic instruments tailored to evaluate and improve the QoL for these patients. Such instruments would enable the identification and comparison of individual care needs and factors influencing their QoL.

**What is already known**

• Penile cancer is a rare malignant neoplasm affecting men globally, and its incidence is rising worldwide.

• Penile cancer is a rare malignant neoplasm affecting men globally, and its incidence is rising worldwide.

• Penile cancer patients' QoL is profoundly affected by various physical symptoms that disrupt their daily life, self-image, and overall life experience.

• No prior review has systematically examined the instruments assessing the QoL of penile cancer patients, and the optimal content of instruments remains unknown.

Alt-text: Unlabelled box

**What this paper adds**

•This review highlights the absence of a psychometrically tested and validated QoL instrument that comprehensively covers all aspects of penile cancer patients' well-being.

• It underscores the need for holistic instruments specifically tailored to evaluate and improve the QoL for penile cancer patients.

• Such instruments would facilitate the identification and comparison of individual care needs and factors that contribute to a more holistic understanding of penile cancer patients' QoL, ultimately improving patient outcomes and care practices.

Alt-text: Unlabelled box

## 1 Background

Penile cancer is a rare malignant neoplasm affecting men globally (Fu et al., 2022; Sung et al., 2021). In 2022, the global incidence of penile cancer was 37,700 newly diagnosed cases, with projections indicating 67,000 cases by 2050 (Ferlay et al., 2020). The incidence of penile cancer is rising worldwide, particularly in European countries; this rise is believed to be associated with increased exposure to human papillomavirus (HPV) and declining rates of childhood circumcision (Fu et al., 2022). A key distinction in clinical practice is between superficial and invasive forms of the disease. Superficial penile cancer is often managed with topical therapies or limited surgical excision and typically has minimal impact on quality of life (Straub Hogan et al., 2022). In contrast, invasive penile carcinoma frequently requires more extensive surgical intervention, including partial or total penectomy, and is associated with significant physical and psychosocial morbidity (Thomas et al., 2021).

Literature highlights the profound emotional impact of diagnosis, treatment, and disfigurement, emphasizing the need for timely care, psychosocial support, and organ-sparing approaches to preserve sexual identity and well-being (Torres Irizarry et al., 2024). Surgery remains the most effective treatment for localized penile cancer (Brouwer et al., 2023; Neuville et al., 2024). However, despite efforts to preserve the organ during surgery, this approach often results in deformities in this sensitive area (Paster and Chipollini, 2022; Törnävä et al., 2022). Additionally, bladder dysfunction and pain are common post-surgery and radiotherapy complications (Witty et al., 2013). Consequently, many men diagnosed with penile cancer experience significant unmet intimacy needs (Paterson et al., 2020), a reduction or cessation of sexual activity (Maddineni et al., 2009), and adverse changes in masculinity and self-esteem (Bullen et al., 2010). The illness and its treatment substantially diminish patients' quality of life (QoL) (Paster and Chipollini, 2022; Törnävä et al., 2022), especially when physical dysfunction occurs (Witty et al., 2013; Harju et al. 2023).

Quality of life means how a person sees and feels about their life, considering their culture, values, goals, and personal expectations (WHO, 2020). Despite the significant psychological burden experienced by many penile cancer patients—up to 50% showing symptoms similar to traumatic stress disorder (Maddineni et al. 2009)—there is currently no standardized or widely accepted tool for measuring QoL in penile cancer population (Harju et al. 2023). In the absence of a comprehensive disease-specific instrument, researchers increasingly adopt a mixed-methods approach—combining qualitative and quantitative tools—to capture the complexity of patient experiences, particularly in rare or sensitive conditions (Klassen et al., 2012). Furthermore, psychosocial resilience—encompassing psychological coping, social support, and the ability to manage practical challenges—has been shown to significantly influence QoL in various cancer populations (Sharpe and Curran, 2006; Zhou et al., 2010). In prostate and testicular cancer, for example, studies have highlighted the importance of emotional adjustment and social connectedness in maintaining QoL, particularly in relation to body image, sexual identity, and treatment-related stress (Paster and Chipollini, 2022; Törnävä et al., 2022; Witty et al., 2013; Harju et al. 2023). These findings suggest that psychosocial resilience is a critical, though often underexplored, component of QoL in penile cancer as well, warranting more focused attention in future research

Therefore, it is evident that penile cancer patients' QoL is profoundly affected by various physical symptoms that disrupt their daily lives, self-image, and overall life experience (Törnävä et al., 2022). Penile cancer patients require enhanced support and counselling to improve their QoL during survivorship, including family and spousal support and the social aspects of life (Torres Irizarry et al., 2024). Healthcare professionals, particularly nurses, are essential in providing support to these patients (Witty et al. 2013). It is especially important to offer individualized support tailored to their specific needs (Harju et al. 2023). Moreover, valid instruments are essential for accurately assessing the factors that affect individuals' lives and coping mechanisms so that healthcare professionals can provide the best possible support to their patients (Soikkeli-Jalonen et al. 2021).

The significant impact of penile cancer on the QoL of affected patients underscores the urgent need to develop comprehensive care strategies. However, no prior review has systematically examined the instruments used to assess the QoL of penile cancer patients. Consequently, the optimal content of these instruments remains unknown. This systematic review aims to fill this critical knowledge gap by providing a comprehensive understanding of the instruments used to assess the QoL of penile cancer patients, thereby informing the development of more effective and targeted instruments to improve their overall well-being.

## 2 Methods

### 2.1 Objective of the study

The aim of this systematic review is to describe the psychometric properties and content of the instruments used for assessing penile cancer patients’ QoL.

Research questions:

1. What instruments have been used to assess the QoL of penile cancer patients?
2. What was the reported validity and reliability of the instruments?
3. What was the content of the instruments?

### 2.2 Study design

A systematic review was chosen to ensure a comprehensive understanding of the limited research topic by incorporating studies that utilized diverse methodologies (Grant and Booth, 2009). Additionally, Bruce et al.’s (2018) systematic approach was adhered to in conducting the review, and the Preferred Reporting Items for Systematic Reviews and Meta-Analyses (PRISMA) guidelines were followed to ensure explicit reporting (Page et al., 2021).

### 2.3 Search strategy

A systematic literature search was conducted in October 2024 across four electronic databases: PubMed, CINAHL, PsycINFO, and the Cochrane Library. An information specialist assisted in the search process. The search utilized the following keywords, their synonyms, and MeSH terms, using Boolean operators: 'penile cancer,' 'quality of life,' and 'instrument': (Penile Neoplasm MeSH OR penile cancer OR penectomy) AND ("quality of life" OR "QoL" OR "life qualit*" OR "Quality of Life"[MeSH]) AND (Surveys and Questionnaires MeSH OR instrument* OR scale* OR test* OR measure* OR tool* OR questionnaire* OR research instrument*). The search was conducted without any time restrictions.

The inclusion criteria were studies that 1) focused on penile cancer patients; 2) studies included at least one instrument specifically developed for assessing quality of life (QoL); 3) described the content of the instrument; 4) were published in scientific peer-reviewed international journals; and 5) were written in English or Finnish. The inclusion criteria were not restricted by study design, allowing for a comprehensive synthesis of the literature. The included studies represented a range of methodological approaches, including quantitative cross-sectional, cohort and longitudinal studies. This diversity enabled a broad understanding of how QoL is assessed among penile cancer patients and what domains are emphasized across different research traditions.

Studies were excluded if they 1) involved groups other than penile cancer patients; 2) did not use an instrument to assess quality of life; 3) were dissertations, editorials, or other non-scientific, non-peer-reviewed articles; or 4) did not meet the language requirements; 5) included only instrument validation without QoL assessment.

### 2.4 Selection process

The selection process followed predefined inclusion and exclusion criteria at all stages. Title and abstract screening were conducted in a single combined phase by two independent reviewers. Full-text assessments were also carried out independently by the same two reviewers. Any disagreements were resolved through discussion until consensus was reached.

**Fig. 1:**
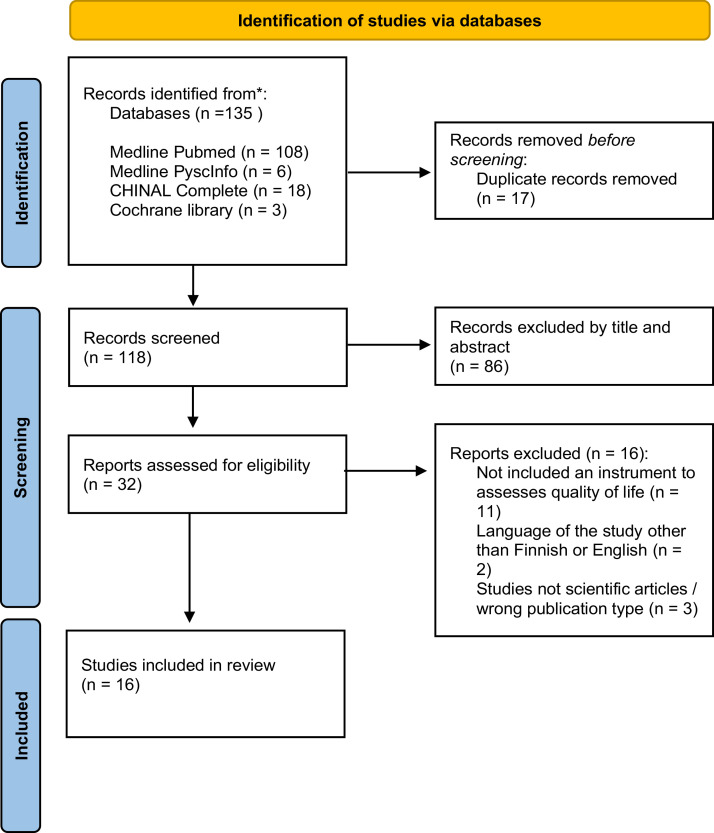
Flow diagram of the literature search following PRISMA (Page et al., 2021).

The initial search yielded 135 articles (Figure 1). After independent title and abstract screening, 32 articles were selected for full-text review. Of these, 11 were excluded for not using a QoL instrument specific to penile cancer patients, two did not meet the language criteria, and three were excluded due to publication type (e.g., not peer-reviewed scientific articles). Ultimately, 16 studies met the inclusion criteria and were included in the review.

### 2.5 Quality appraisal

For the critical appraisal of the study quality, the Joanna Briggs Institute's (JBI) checklist for analytical cross-sectional studies (Moola et al., 2017) and the checklist for cohort studies (Moola et al., 2024) were utilized. The studies were scored using a binary system: 'yes' received one point, while 'no' and 'unclear' received zero points (Table 1). The quality appraisal was conducted independently by two authors, followed by a discussion to reach a consensus on the quality scores. The overall quality of the studies was deemed good, with all cross-sectional studies being of high quality and the cohort study being of medium quality (Table 1).

**Table 1:** Critical appraisal of included studies.

**Cross-sectional studies**												
***Study***	***1. Clear inclusion criteria***	***2. Detailed subject and study description***	***3. Valid/ reliable measured exposure***	***4. Objective, standard criteria used for measurement***		***5.Cofounding factors identified***	***6.Cofounding factor strategy***		***7. Valid/ reliable outcome measure***	***8.Appropriate statistical analysis***		***Quality (JBI)***
Jovanovic et.al 2023	1	1	1	1		1	0		1	1		7/8
Firmansyah et.al 2023	1	1	1	1		0	0		1	1		6/8
Jakobsen et.al 2022	1	1	1	1		1	0		1	1		7/8
Branney et.al 2022	1	1	1	1		0	0		1	1		6/8
Blinded for review et.al 2021	1	1	1	1		1	0		1	1		7/8
Croghan et.al 2021	1	1	1	1		1	0*		1	1		7/8
Pérez et.al 2020	1	1	1	1		1	0		1	1		7/8
Suarez-Ibarrola et.al 2018	1	1	1	1		1	0		1	1		7/8
Gambachidze et.al 2018	1	1	1	1		1	0		1	1		7/8
Draeger et.al 2018	1	1	1	1		0	0		1	1		6/8
Wan et.al 2018	1	1	1	1		1	0*		1	1		7/8
Sosnowski et.al 2017	1	1	1	1		1	0		1	1		7/8
Sosnowski et.al 2016	1	1	1	1		1	0		1	1		7/8
Kieffer et.al 2014	1	1	1	1		1	0		1	1		7/8
Gulino et.al 2013	1	1	1	1		1	0		1	1		7/8
**Cohort studies**												
***Study***	***1. Similar groups recruited from the same population***	***2. Exposures measured similarly in both groups***	***3. Valid and reliable measured exposure***	***4.Confounding factors identification***	***5.Strategies to deal with confounding factors stated***	***6.Participants free of outcome at the start of the study (or at the moment of exposure)***	***7. Valid and reliable outcome measure***	***8. Adequate follow up time***	***9. Completed follow up. Loss reasons reported.***	***10.Incomplete follow up strategy***	***11. Appropriate statistical analysis***	***Quality (JBI)***
Warli et.al 2023	1	1	1	0	0	0	1	1	1	0	0	6/11

Abbreviations for scoring cross-sectional and cohort study appraisal; 1 = yes, 0 = no, 0*=unclear.

### 2.6 Data collecting and analysis process

The initial data extraction focused on collecting, summarizing, and tabulating key information from the included studies, guided by the research questions (Aromataris and Pearson, 2014). One researcher independently extracted data from each included study using a structured data extraction form, based on a plan developed collaboratively by the research team. Any uncertainties or ambiguities encountered during the process were discussed and resolved in consultation with the team to ensure consistency and accuracy. The extracted data included authors, year of publication, study objectives, design, participant characteristics, data collection instruments, and analysis methods (Table 2). Any discrepancies between reviewers were resolved through discussion and consensus. The tabulated information was synthesized using descriptive synthesis (Popay et al., 2006).

**Table 2:** Study characteristics.

Author(s), Year of publication, Country	Objective of the study	Study design	Number of participants, Age (mean)	Surgical treatment	Data collection instruments	Outcomes	Recall period	Conditions of administration	Analysis
Gulino et.al 2013, Italy	To assess psychological and sexual outcomes and the quality of life	Three-point longitudinal study	n=42 Mean age 56	Glansectomy, partial penectomy with urethral glanduloplasty	Bigelow-Young questionnaire* IIEF-15^i^	QOL, sexual function	NR	Participants were interviewed individually	t-test ANOVA
Kieffer et.al 2014, Netherlands	To assess the impact of primary surgery on sexuality and health related quality of life.	Cross-sectional study	n=90 Mean age 65.4	Laser/local excision with or without circumcision, glans amputation with or without reconstruction, vs penectomy or partial penectomy	SF-36^*,ii^ IOCv2^*,iii^ IIEF-15^i^	QOL, sexual function, urinary function	NR	NR	ANOVA/ANCOVA.
Sosnowski et al. 2016, Poland	To assess quality of life	Cross-sectional study	n=10 Mean age 60.5	Total penectomy with perineal urethrostomy	EORTC QLQ C-30^*,iv^ RSES^v^ CMNI^vi^ IIEF-15^i^ IPSS^vii^	QOL, sexual function, self-esteem, masculinity, urinary function	NR	Self-administered questionnaires	Mann-Whitney U-test
Sosnowski et al. 2017, Poland	To compare aspects of QoL	Cross-sectional study	n=51 Mean age 62.9	Local excision or partial penectomy or total penectomy	QLQ C-30*^iv^	QOL	NR	Self-administered mailed questionnaires	Spearman chi-squared test.
Suarez-Ibarolla et al. 2018, Mexico	To assess the impact of primary surgery on health-related quality of life (HRQoL) and sexual function	Cross-sectional study	n=10 Mean age 54.3	Partial or total penectomy, inguinal lymphadenectomy	SF-36*^ii^ IIEF-5i	HRQOL, sexual function	NR	Self-administered questionnaires	Mann-Whitney U-test
Gambachidze et.al 2018, France	To assess the long-term functional results of organ-preserving brachytherapy	Cross-sectional study	n=23 Mean age 63.4	Brachytherapy	EQ-5D-3L^*,viii^ ICSmaleSF^ix^ IIEF-15^i^ IMGI^x^	QOL, urinary function, sexual function	NR	Self-administered mailed questionnaires	Chi-squared test, Mann-Whitney U-test, Kruskall-Wallis, t-test
Draeger et.al 2018, Germany	To apply a validated questionnaire (EORTC QLQ-C30) to reference data of the general population and to develop a new unvalidated questionnaire HRO-PE29	Cross-sectional study	n=76 Mean age 63.9	Local surgical	EORTC QLQ-C30*^iv^ HRO-PE29^*,xi^	QOL	NR	Self-administered at a preadmission examination or on the day of admission for in-patient treatment.	Descriptive statistics
Wan et.al 2018, China	To comparatively assess the quality-of-life parameters	Cross-sectional study	n=15 Mean age 62	Partial penectomy or wide local excisions	EORTC-QLQ-C30*^iv^ IIEF-15^1^ SEAR^xii^ EDITS^xiii^	HRQOL, sexual function, urinary function	NR	NR	t-test
Perez et.al 2020, Colombia	To describe oncological and functional outcomes	Cross-sectional study	n=32 Mean age 55.1	Reconstructive organ-sparing surgery (OSS)	EQ-5D-3L*^viii^ ICIQ-MLUTS^xiv^ IIEF-5^i^	QOL, urinary symptoms, sexual function	NR	Phone interviews	Kaplan-Meier plots, Fisher’s exact test.
Croghan et.al 2021, Ireland	To assess patient-reported outcomes	Cross-sectional study	n=35 Mean age 61	Partial glansectomy, radical glansectomy with neo glans formation, or partial penectomy-with reconstruction	EORTC QLQ-C30*^iv^ IMGI^vi^ IIEF-5^i^	QOL, urinary, erectile and sensory function, perceived genital appearance	NR	Self-administered mailed questionnaires	Mann-Whitney U-test, mean score
Blinded for review et.al 2021, Finland	To report treatment-related HRQoL changes and the specific deficits that have the most profound effect, and to identify patient-related factors that predispose to a worse perceived HRQoL.	Cross-sectional study	n=68 Mean age 70	Penile resection or penectomy or minor surgery	15D^*,xv^ RSES^v^ OSFQ^xvi^ EHS^xvii^	HRQOL, self-esteem, overall sexual functioning, erections, change in sexual function	NR	Self administered mailed questionnaires	Mann-Whitney U-test, Kruskal-Wallis, Spearman
Branney et.al 2022, UK	To assess the feasibility of the MGSIS-5xviii and the G3L-20xix for measuring quality of life.	Cross-sectional study	n=22 no demographic information was collected	Sentinel node biopsy only or inguinal node dissection only or inguinal node dissection with postoperative pelvic radiotherapy with or without chemotherapy	EORTC QLC-C30*^iv^ IIEF-5^i^ MGSIS-5^xviii^ G3L-20^xix^ mLGUCQ^xviii^	QOL, genital body image, sexual function, lymphedema	NR	Self administered mailed questionnaires	Descriptive statistics
Jakobsen et.al 2022, Denmark	To assess prevalence of voiding, sexual symptoms and quality of life	Cross-sectional study	Group 1 (at diagnosis) n=51, mean age 70.3 Group 2 (1 year after diagnosis) n=69, mean age 67.2 Group 3 (2 years after diagnosis) n=37, mean age 66.4	Local resection and/or laser, partial penectomy, total penectomy	Unnamed study- specific questionnaire*	QOL, voiding and sexual function	NR	Self-administered during the clinic visit or self-administered mailed questionnaire	ANOVA, nonparametric tests (not reported)
Warli et.al 2023, Indonesia	To assess survival and quality of life	Cohort study	n=20 Mean age (early resection) 42.2. Mean age (Neoadjuvant chemotherapy) 44.2	Histologically confirmed penile cancer with bulky lymph nodes	EORTC QLQ-C30*^iv^	QOL	NR	Interviews by mail or phone or self-administered during clinic visits	Mean values Kaplan-Meier
Firmansyah et.al 2023, Indonesia	To assess health-related quality of life	Single-Center Cross-sectional study.	n=9 Mean age 54.44	Partial penectomy, total penectomy	EORTC QLQ-C30*^iv^	QOL	NR	NR	Descriptive statistics
Jovanovi´c et. al 2023, Serbia	Evaluate PC patients’ post-treatment quality of life (QoL), sexual activity, self-esteem, fatigue and fear of disease recurrence	Cross-sectional study	n=31 Mean age 66.9	Histopathologically confirmed diagnosis of penile cancer	EORTC QLQ-C30*^iv^ IIEF-15^i^ RSES^v^ HADS^xx^ MFI^xxi^ FCRI^xxii^	QOL, sexual activity, self-esteem, fatigue and fear of disease recurrence	NR	Questionnaires filled during routine outpatient visit	Descriptive statistics, Pearson and Spearman, hierarchical linear regression analysis

Abbreviations:

* Instrument used for Quality-of-Life assessment

NR=not reported

^i^ The International Index of Erectile Function Questionnaire

^ii^ 36-Item Short Form Health Survey*

^iii^ Impact of Cancer Version 2*

^iv^ European Organisations for Research and the Treatment of Cancer Quality of Life Questionnaire*

^v^ Rosenberg Self-Esteem Scale

^vi^ Conformity to Masculine Norms Inventory

^vii^ International Prostate Symptom Score

^viii^ EuroQol 5-dimension three-level questionnaire*

^ix^ International Continence Society male Short form questionnaire + Peelings voiding picture

^x^ Index of Male Genitalia Image

^xi^ Quality of life Questionnaire Penile Cancer Rostock*

^xii^ Self-esteem and Relationship Questionnaire

^xiii^ Erectile Dysfunction Inventory of Treatment Satisfaction Questionnaire

^xiv^ International Consultation on Incontinence Modular Questionnaire for Male Lower Urinary Tract Symptoms

^xv^ 15 Dimensions of Health Related Quality of Life*

^xvi^ Overall Sexual Functioning Questionnaire

^xvii^ Erection Hardness Score

^xviii^ Male Genital Self-Image Scale

^xix^ Groin and Lower Limb Lymphedema questionnaire

^xx^ Hospital Anxiety and Depression scale

^xxi^ Multidimensional Fatigue Inventory

^xxiixxii^ Fear of Cancer Recurrence Inventory

Additionally, all QoL instruments used in the studies were identified and tabulated (Table 3), including details on instrument type, domains, scaling, and reported validity and reliability.

**Table 3:** Instruments used to assess penile cancer patients’ quality of life.

**Generic instruments**					
**Name of the Instrument**	**Reference**	**Domains**	**Items**	**Scale**	**Validity / Reliability**
Bigelow-Young Quality of life questionnaire	Gulino et.al 2013	1. Physical condition of home (6 items) 2. Total satisfaction in home (4 items) 3. Responsibility for self & home (6 items) 4. Self and home maintenance (3 items) 5. Employment success (4 items) 6. Meaningful use of time (3 items) 7. Psychiatric distress (3 items) 8. Psychological Well-being (5 items) 9. Interpersonal relations (5 items)	38	NR	Reported good face validity and reported to be sensitive / Reliability NR
36-Item Short Form Health Survey (SF-36)	Kieffer et.al 2014 Suarez-Ibarolla et. al 2018	I Physical component summary score (content NR)II Mental component summary score (content NR) 1. physical functioning 2. role-physical 3. role-emotional 4. bodily pain 5. social functioning 6. mental health 7. vitality 8. general health.	36	0-100 All items linearly converted to a 0 to 100 scale, higher scores representing better functioning.	NR
EuroQol 5-dimension three-level questionnaire (EQ-5D- 3 L)	Gambachidze et.al 2018 Perez et.al 2020	Aspect of health (part I) and The Overall Health Status (part II)	Part I: 1. Mobility 2. Self-care 3. Usual activities 4. Pain or discomfort 5. Anxiety and depression Part II: The Overall Health Status	Part I: Level 1 indicating no problems, Level 2 some problems, Level 3 extreme problems. Part II: Visual Analog Scale (EQ-VAS score) 0-100 (0= the worst Health imaginable –100= the best heath imaginable)	Validated in previous studies/ Reliability NR
15 Dimensions of Health Related Quality of Life (15D)	Blinded for review et.al 2021	No separate domains	1. Mobility 2. Vision 3. Hearing 4. Breathing 5. Sleeping 6. Eating 7. Speech 8. Excretion 9. Usual activities 10. Mental function 11. Discomfort and symptoms 12. Depression 13. Distress 14. Vitality 15. Sexual activity	0-5 Higher score indicates a higher HRQoL.	Validated in previous studies / Reliability NR
**Cancer specific instruments**					
**Name of the Instrument**	**Reference**	**Domains**	**Items**	**Scale**	**Validity / Reliability**
Impact of Cancer Version 2 (IOC)	Kieffer et.al 2014	I domain - Positive impact (4 items) II domain - Negative impact (4 items) Additional items (2)	I domain 1. Altruism and empathy 2. Health awareness 3. Meaning of cancer 4. Positive self-evaluation II domain 5. Appearance concerns6. Body change concerns7. Life interferences8. WorryAdditional items 9. Employment10. Relationship concerns	In positive impact domain a high score represents stronger agreement/positive response. In negative impact domain a high score indicates a more negative response.	NR
European Organisations for Research and the Treatment of Cancer Quality of Life Questionnaire (EORTC QLQ C-30)	Sosnowski et.al 2016 Sosnowski et.al 2017 Draeger et.al 2018 Wan et.al 2018 Croghan et.al 2021 Branney et.al 2021 Warli et.al 2023 Firmansyah et.al 2023 Jovanovic et.al 2023	Part I Functional domains 1. physical 2. role 3. cognitive 4. emotional 5. social Part II Symptom domains 1. fatigue 2. nausea and vomiting 3. pain 4. dyspnea 5. insomnia 6. appetite loss 7. constipation 8. diarrhoea 9. financial problems Part III Global health status 1. overall health past week 2. overall quality of life past week	30	For functional and symptom domains a four-point Likert scale is presented (1=never, 2=sometimes, 3=often and 4=very often). For Global Health Status a Likert scale (1=very poor and 7=excellent). Higher global health status and functioning scores combined with lower symptom scores represent a better QoL.	Psychometrically tested (tests not reported), Validated in previous studies. Validated translations.
**Penile cancer specific instruments**					
**Name of the Instrument**	**Reference**	**Domains**	**Items**	**Scale**	
Unnamed study specific questionnaire	Jakobsen et al. 2022	1. Introductory questions 2. Quality of life 3. Dejection and worry 4. Urinary tract 5. Sex life 6. Health in general	19 Introductory questions 8 Quality of life items 8 Dejection and worry items 10 Urinary tract items 21 Sex life items 14 Health in general items	Quality of life (No quality of life–The best possible quality of life) Dejection and worry (No, never–Yes, every night) Urinary tract (Never–Always) Sex life (altering answer options) Health in general (No–Yes)	Face validity assessed in a pilot study / The questionnaire was pilot tested by 6 PC patients
Quality of life Questionnaire Penile Cancer Rostock (HRO-PE29)	Draeger et.al 2018	1. Adverse effects of systemic treatment 2. Lymphedema 3. Alopecia 4. Voiding 5. Sexual functioning 6. Sexual pleasure 7. Future prospects 8. Genital symptoms 9. Body image	29	Four possible answers (not at all, least, moderate, very).	Unvalidated / Reliability NR

NR=not reported.

The study utilized an inductive, data-driven qualitative content analysis, in which the coding categories were derived directly from the data without relying on pre-existing theoretical frameworks. An inductive content analysis of the instruments was then conducted independently by two authors and subsequently reviewed and confirmed by the full research team (Elo and Kyngäs, 2008). The analysis involved extracting the content of each instrument, grouping similar domains into subcategories, and then synthesizing them into broader categories (Table 4).

**Table 4:** Content of instruments used to assess penile cancer patients QoL.

Main theme	Theme	Subtheme	Included in instruments
**Assessment of physical functioning**	Physical capabilities	Physical performance	EORTC QLQ C-30^xxii^ EQ-5D-3L^xxii^ SF-36^xxii^ 15D^xxii^
		Physical Abilities	EQ-5D-3L^ii^ 15D^iv^ USQ^xxii^ HRO-PE29^xxii^
	Secondary effects of penile cancer and its treatments	Pain and discomfort	EORTC QLQ C-30^i^ EQ-5D-3L^ii^ SF-36^iii^ 15D^iv^
		Adverse symptoms of cancer treatment	EORTC QLQ C-30^i^ HRO-PE29^vi^
		Voiding function	EORTC QLQ C-30^i^ 15D^iv^
**Assessment of psychosocial resilience**	Psychological well-being	Emotional functioning	EORTC QLQ C-30^i^ SF-36^iii^ 15D^iv^ USQ^v^ Bigelow-Young QoL questionnaire
		Mental burden	EQ-5D-3L^ii^ 15D^iv^ USQ^v^ IOC Bigelow-Young QoL questionnaire^xxii^
		Self-image change	USQ^v^ HRO-PE29^vi^ IOC^viii^
		Meaningfulness of life	SF-36^iii^ USQ^v^ Bigelow-Young QoL questionnaire^vii^
	Social resources	Meaningful relations	Bigelow-Young QoL questionnaire^vii^ IOC^viii^
		Social functioning	EORTC QLQ C-30^i^ SF-36^iii^
	Financial and domestic coping	Financial changes	EORTC QLQ C-30^i^ HRO-PE29^vi^ Bigelow-Young QoL questionnaire^vii^ IOC^viii^
		Satisfaction for home maintenance	Bigelow-Young QoL questionnaire^vii^
**Assessment of overall life functions**	Overall quality of life	Long-term QoL	EORTC QLQ C-30^i^ USQ^v^
		Short term QoL	EORTC QLQ C-30^i^ USQ^v^
	Health-related quality of life	Assessment of overall health	EORTC QLQ C-30^i^ EQ-5D- 3L^ii^
		Health awareness	SF-36^iii^ IOC^viii^

^i^ Sosnowski et al. 2016, Sosnowski et al. 2017, Draeger et al. 2018, Wan et al. 2018, Croghan et al. 2021, Branney et al. 2022, Warli et al. 2023, Firmansyah et al. 2023, Jovanovic et al. 2023.

^ii^ Gambachidze et al. 2018, Perez et al. 2020.

^iii^ Kieffer et al. 2014, Suarez-Ibarolla et al. 2018.

^iv^ Blinded for review et al. 2021.

^v^ Jakobsen et al. 2022.

^vi^ Draeger et al. 2018.

^vii^ Gulino et al. 2013.

^viii^Kieffer et al. 2014.

## 3 Results

### 3.1 Description of the studies

The results of this systematic review are based on 16 studies conducted between 2013 and 2023 (Table 2), half of which (n=8) were completed in the 2020s. The selected studies were conducted in Poland (Sosnowski et al., 2017, 2016), Indonesia (Firmansyah et al., 2023; Warli et al., 2023), Italy (Gulino et al., 2013), the Netherlands (Kieffer et al., 2014), Mexico (Suarez-Ibarrola et al., 2018), France (Gambachidze et al., 2018), Germany (Draeger et al., 2018), China (Wan et al., 2018), Colombia (Perez et al., 2020), Ireland (Croghan et al., 2021), Finland (Harju et al., 2021), the United Kingdom (Branney et al., 2022), Denmark (Jakobsen et al., 2022), and Serbia (Jovanović et al., 2023). The studies were published in medical journals (n=14) and nursing journals (n=2). One study was a three-point longitudinal study (Gulino et al., 2013), one was a cohort study (Warli et al., 2023), and the remaining were cross-sectional studies.

The studies’ sample sizes ranged from 9 to 76 participants, and the participants' ages ranged from 42 to 70 years. The penile cancer patients received various treatments, most commonly local resection, glansectomy, partial or total penectomy with perineal urethrostomy, and lymphadenectomy. Additionally, reconstructive surgery was performed in two studies (Kieffer et al., 2014; Perez et al., 2020). One study focused on the effects of brachytherapy on the QoL of penile cancer patients (Gambachidze et al., 2018), while another examined the connection between sentinel node biopsy, inguinal node dissection, and postoperative pelvic radiotherapy with or without chemotherapy, and their impact on the perceived QoL of penile cancer patients (Branney et al., 2022) (Table 2).

### 3.2 Instruments used to assess penile cancer patients’ QoL

Based on the review, eight instruments were identified, of which four were generic, two were cancer-specific, and two were penile cancer-specific (Table 3). The instruments were used either individually, combined together, or in conjunction with other instruments addressing various symptoms or issues associated with living with cancer (Table 2).

Although the search strategy was designed to identify instruments assessing quality of life (QoL) across the full spectrum of penile cancer types and treatment modalities, the available literature predominantly focused on patients undergoing invasive interventions. QoL assessments were largely limited to outcomes following partial or total penectomy, with a notable absence of validated instruments or data addressing patients treated with conservative or non-invasive approaches (Table 3).

None of the included studies reported the recall period of the instruments used (Table 2). The questionnaires were most commonly self-administered, either during clinic visits or sent by post (Branney et al., 2022; Croghan et al., 2021; Draeger et al., 2018; Gambachidze et al., 2018; Harju et.al 2021; Jakobsen et al., 2022; Sosnowski et al., 2016; 2021; Suarez-Ibarolla et al. 2018). Some studies used face-to-face or telephone interviews (Gulino et al., 2013; Perez et al., 2020; Warli et al., 2023). In three studies, the mode of administration was not reported (Firmansyah et al., 2023; Kieffer et al., 2014; Wan et al., 2018).

Four generic QoL instruments – Bigelow-Young Quality of Life Questionnaire (Gulino et al., 2013), 36-Item Short Form Health Survey (SF-36) (Kieffer et al., 2014 ; Suarez-Ibarolla et al., 2018), EuroQol 5-Dimension Three-Level Questionnaire (EQ-5D-3 L) (Gambachidze et al., 2018; Perez et al., 2020), and 15 Dimensions of Health-Related Quality of Life (15D) (Harju et al., 2021) – had identifiable common domains, such as the assessment of physical and mental symptoms and functional capacity (Table 3). Generic instruments, when reported, included 15–38 items evaluating various areas of QoL. Typically, these instruments evaluated both physical and mental aspects of life, assessing their realization on a general level, including physical and mental functioning and coping with daily life activities (Table 3).

The two cancer-specific instruments – the Impact of Cancer Version 2 (IOC) (Kieffer et al., 2014) and the European Organisation for Research and the Treatment of Cancer Quality of Life Questionnaire (EORTC QLQ-C30) (Sosnowski et al., 2016; Sosnowski et al., 2017; Draeger et al., 2018; Wan et al., 2018; Croghan et al., 2021; Branney et al., 2021; Warli et al., 2023; Firmansyah et al., 2023; Jovanovic et al., 2023) – differed in structure and focus. The IOC assessed both the positive and negative impacts of cancer, as well as the social and financial burdens or impacts associated with the disease. In contrast, the EORTC QLQ-C30 was structured into three distinct domains: one evaluating comprehensive functional capabilities, another covering a broad range of symptoms, and a third assessing global health status (Table 3).

Two instruments were specific to penile cancer. An unnamed, study-specific questionnaire assessed inventory questions, quality of life, dejection and worry, urinary tract health, sex life, and general health in penile cancer patients (Jakobsen et al., 2022). Additionally, Draeger et al. (2018) developed the Quality-of-Life Questionnaire for Penile Cancer Rostock (HRO-PE29) in their study, which comprised nine domains: adverse effects of systemic treatment, lymphedema, alopecia, voiding, sexual functioning, sexual pleasure, future prospects, genital symptoms, and body image (Table 3).

### 3.3 The validity and reliability of the instruments

The validity of the instruments was relatively underreported: only two studies provided descriptions, and three did not report on validity at all. When validity was reported, it was mostly limited to face validity or pilot testing. Moreover, for instruments described as valid, the validation was reported solely in previous studies. Furthermore, reliability was not adequately reported in any of the studies.

The Bigelow-Young Quality of Life instrument was reported to have good face validity as well as high sensitivity (Gulino et al., 2013), and the validity of the EQ-5D-3 L and 15D instruments has been confirmed by previous studies (Gambachidze et al., 2018; Harju et al., 2021; Perez et al., 2020). However, the validity of the generic SF-36 instrument was not reported (Kieffer et al., 2014; Suarez-Ibarrola et al., 2018) (Table 3).

The cancer-specific EORTC QLQ-C30 was reported to be psychometrically tested and validated in previous studies, and it has validated translations in several languages. In contrast, the validity of the IOC instrument was not reported (Table 3).

The face validity of an unnamed study-specific questionnaire was tested in a pilot study with six penile cancer patients, while the HRO-PE29 was not tested for validity (Draeger et al., 2018; Jakobsen et al., 2022). In the other studies included in the systematic review, the reliability of the instruments was not reported (Table 3).

### 3.4 Content of the instruments

To structure the content analysis of the QoL instruments, three overarching domains were identified based on the content of the included studies: (1) physical functioning, (2) psychosocial resilience, and (3) overall life functioning (Table 3).

#### 3.4.1 Assessment of physical functioning

The assessment of physical functioning in the included QoL instruments encompassed two main components: (1) physical capabilities and (2) secondary effects of penile cancer and its treatments. These components were identified based on the content of the instruments and are detailed in Table 4.

Physical capabilities involved the assessment of physical performance and physical abilities, while physical performance evaluation focused on the overall physical functioning of the patient (Sosnowski et al., 2016; Sosnowski et al., 2017; Draeger et al., 2018; Wan et al., 2018; Croghan et al., 2021; Branney et al., 2022; Warli et al., 2023; Firmansyah et al., 2023; Jovanovic et al., 2023; Gambachidze et al., 2018; Perez et al., 2020; Kieffer et al., 2014; Suarez-Ibarolla et al., 2018; Harju et al., 2021). The instruments evaluated physical abilities through self-care (Gambachidze et al., 2018; Perez et al., 2020), physical coping assessment (Jakobsen et al., 2022; Harju et al., 2021), and sexual function (Draeger et al., 2018; Harju et al., 2021).

The instruments also identified the secondary effects of penile cancer and its treatments (Table 4). Pain and discomfort experienced by penile cancer patients were included in the instruments evaluating QoL (Sosnowski et al., 2016; Sosnowski et al., 2017; Draeger et al., 2018; Wan et al., 2018; Croghan et al., 2021; Branney et al., 2022; Warli et al., 2023; Firmansyah et al., 2023; Jovanovic et al., 2023; Gambachidze et al., 2018; Perez et al., 2020; Kieffer et al., 2014; Suarez-Ibarolla et al., 2018; Harju et al., 2021). The assessment of adverse symptoms of cancer treatments, such as genital symptoms or nausea, was also included (Sosnowski et al., 2016; Sosnowski et al., 2017; Draeger et al., 2018; Wan et al., 2018; Croghan et al., 2021; Branney et al., 2022; Warli et al., 2023; Firmansyah et al., 2023; Jovanovic et al., 2023; Draeger et al., 2018). Additionally, voiding function was evaluated (Sosnowski et al., 2016; Sosnowski et al., 2017; Draeger et al., 2018; Wan et al., 2018; Croghan et al., 2021; Branney et al., 2022; Warli et al., 2023; Firmansyah et al., 2023; Jovanovic et al., 2023; Harju et al., 2021).

#### 3.4.2 Assessing psychosocial resilience

Psychosocial resilience was defined as a multidimensional construct encompassing psychological well-being, social resources, and financial and domestic coping (Table 4).

The evaluation of psychological well-being in the instruments was based on assessing emotional functioning (Sosnowski et al., 2016; Sosnowski et al., 2017; Draeger et al., 2018; Wan et al., 2018; Croghan et al., 2021; Branney et al., 2022; Warli et al., 2023; Firmansyah et al., 2023; Jovanovic et al., 2023; Kieffer et al., 2014; Suarez-Ibarrola et al., 2018; Harju et al., 2021; Jakobsen et al., 2022; Gulino et al., 2013) and mental burden (Gambachidze et al., 2018; Perez et al., 2020; Harju et al., 2021; Jakobsen et al., 2022; Kieffer et al., 2014; Gulino et al., 2013). This assessment included the evaluation of perceived self-image change (Jakobsen et al., 2022; Draeger et al., 2018; Kieffer et al., 2014) and perceived meaningfulness of life (Kieffer et al., 2014; Suarez-Ibarrola et al., 2018; Jakobsen et al., 2022; Gulino et al., 2013).

Social resources included assessment of meaningful relations (Gulino et al., 2013; Kieffer et al., 2014) and social functioning (Sosnowski et al., 2016, Sosnowski et al., 2017, Draeger et al., 2018, Wan et al., 2018, Croghan et al., 2021, Branney et al., 2022, Warli et al., 2023, Firmansyah et al., 2023, Jovanovic et al., 2023; Kieffer et al., 2014, Suarez-Ibarolla et al., 2018).

The evaluation of financial and domestic coping included financial changes (Sosnowski et al., 2016, Sosnowski et al., 2017, Draeger et al., 2018, Wan et al., 2018, Croghan et al., 2021, Branney et al., 2022, Warli et al., 2023, Firmansyah et al., 2023, Jovanovic et al., 2023; Draeger et al., 2018; Gulino et al., 2013; Kieffer et al., 2014) and satisfaction regarding home maintenance (Gulino et al., 2013).

#### 3.4.3 Assessment of overall life functions

The assessment of overall life functioning in the included QoL instruments focused on two key aspects: (1) overall quality of life and (2) health-related quality of life (Table 4).

Overall QoL involved assessing both long- and short-term QoL (Sosnowski et al., 2016; Sosnowski et al., 2017; Draeger et al., 2018; Wan et al., 2018; Croghan et al., 2021; Branney et al., 2022; Warli et al., 2023; Firmansyah et al., 2023; Jovanovic et al., 2023; Jakobsen et al., 2022), while health-related QoL included the assessment of overall health (Sosnowski et al., 2016; Sosnowski et al., 2017; Draeger et al., 2018; Wan et al., 2018; Croghan et al., 2021; Branney et al., 2022; Warli et al., 2023; Firmansyah et al., 2023; Jovanovic et al., 2023; Gambachidze et al., 2018; Perez et al., 2020) and health awareness (Kieffer et al., 2014; Suarez-Ibarrola et al., 2018).

## 4 Discussion

This systematic review examined the content and psychometric properties of instruments used to assess the QoL of penile cancer patients. Notably, the literature search revealed very few studies on this topic, underscoring a significant gap in research. The limited number of studies identified and included in this review highlights the need for greater attention to the QoL of penile cancer patients.

The findings of this review indicate that existing QoL research in penile cancer has predominantly focused on patients undergoing invasive treatments. This emphasis is understandable, given the significant physical, psychological, and sexual consequences associated with procedures such as partial or total penectomy (Thomas et al., 2021). However, the literature reveals a notable lack of QoL data concerning patients with superficial disease, who are typically treated with topical therapies or limited surgical excision (Straub Hogan et al., 2022). Although these patients may not experience the same degree of physical morbidity, their psychosocial experiences, concerns about recurrence, and treatment burden remain clinically relevant. These findings underscore the need for future research to include conservatively treated patient populations and to develop or adapt quality of life instruments that reflect the full spectrum of disease presentation and management in penile cancer.

Furthermore, eleven out of the 16 identified studies were conducted in Europe. Notably, there were no studies from North America, Australia, Oceania, or Africa. Although penile cancer is becoming more common in European countries, its incidence is also rising worldwide (Fu et al., 2022), which underscores the need for increased global awareness and research. Penile cancer patients experience disruptive physical symptoms and changes in self-image (Törnävä et al., 2022), which can be presumed to be universal among men. Therefore, a broader perspective on the QoL of these patients would help in understanding regional differences and how cultural contexts affect patient experiences and outcomes, leading to more culturally sensitive care. Additionally, different healthcare systems can impact the QoL of patients differently (Paterson et al., 2020), and studies from various regions can help identify best practices and areas for improvement in patient care.

To evaluate the QoL among penile cancer patients, this review identified eight QoL instruments with varying contents, of which only two were specifically developed for the penile cancer population (Draeger et al., 2018; Jakobsen et al., 2022). The most frequently utilized instrument across the studies was the cancer-specific EORTC QLQ-C30, which assesses QoL across multiple life domains but does not encompass aspects such as mental burden, life meaningfulness, or changes in self-image. Additionally, the selection of QoL instruments in clinical practice should consider the type of treatment (e.g., conservative vs. radical surgery), the phase of care (e.g., diagnosis, treatment, follow-up), and the patient's physical and psychological status. For example, patients undergoing partial or total penectomy may benefit from instruments that emphasize body image, sexual function, and emotional well-being, whereas those treated conservatively may require tools that capture anxiety related to recurrence and treatment burden. Tailoring instrument selection to the clinical context could enhance the relevance and sensitivity of QoL assessments. Overall, none of the instruments provided a comprehensive assessment of the patients' QoL, potentially leading to an incomplete understanding of their experiences and needs. To overcome the limitations in content coverage and comprehensively address the diverse symptoms experienced by penile cancer patients, 14 studies in this review employed a combination of instruments. The findings of this review indicate that the current QoL metrics are not sufficiently comprehensive for measuring the QoL in this patient group.

While many of the instruments acknowledged the importance of changes in physical functioning, only two mentioned sexual function (Draeger et al., 2018; Harju et al., 2021). This limitation is noteworthy, as it restricts their holistic perspective and can result in inadequate support related to intimacy and sexual health, which are crucial for the well-being of these patients. Moreover, problems with intimacy and sexual activity are among the most significant issues for men diagnosed with penile cancer (Maddineni et al., 2009; Paterson et al., 2020; Sakalis et al., 2022). Psychosexual and relational aspects are central to the QoL of penile cancer patients but remain insufficiently addressed in existing instruments. Adapting and validating such instruments could be a feasible and efficient strategy to address this gap.

Additionally, some of the studies reported QoL instruments that identified psychosocial resilience as part of the QoL concerning expressed emotions or mental burden. However, changes in body image or self-esteem and the assessment of meaningful relationships or social functioning were rarely included. It is likely that penile cancer treatments affect interpersonal relationships, and the psychological impact of disfigurement, particularly the emotional distress it can cause, must not be overlooked (Paster and Chipollini, 2022). Furthermore, understanding patients' social resources should include the impact of penile cancer on partners (Paterson et al., 2020; Torres Irizarry et al., 2024). Nevertheless, social resources were included in only a few instruments, and the aspect of spousal or partner support was not reported in any of the QoL instruments or studies. For a holistic perspective, these factors need to be addressed in comprehensive instruments assessing the QoL of penile cancer patients.

The validity and reliability of the instruments included in this review were reported in varying ways, mostly inadequately. The most frequently used instrument, the EORTC QLQ-C30, was psychometrically tested and validated in several languages in previous studies. Additionally, two instruments were reported to have been validated in prior research (Gambachidze et al., 2018; Harju et al., 2021; Perez et al., 2020). However, the psychometric properties of the other instruments were sparsely reported, with only pilot testing (Jakobsen et al., 2022) and sensitivity and face validity (Gulino et al., 2013), or lacked validity or reliability reporting at all (Kieffer et al., 2014; Suarez-Ibarolla et al., 2018; Draeger et al., 2018). Only three instruments reported sufficient validation – two generic QoL instruments and one cancer-specific. However, neither of the penile cancer-specific instruments were psychometrically tested. The need for a validated penile cancer-specific QoL instrument has been identified in prior studies (Maddineni et al., 2009; Törnävä et al., 2022); this observation is further supported by the results of this review, which did not identify any penile cancer-specific instrument with comprehensively reported psychometric properties. Neither generic nor cancer-specific instruments account for all aspects of the specific needs of penile cancer patients, which is crucial for identifying and addressing their unique needs (Paster and Chipollini, 2022; Törnävä et al., 2022; Harju et al., 2023). In rare cancers, where disease-specific QoL tools are lacking, recent literature supports adapting existing instruments and using mixed-methods approaches to enhance the relevance and depth of assessment (Klassen et al., 2012). Though, those approaches are not easy to adapt in clinical practice.

Clinically, the lack of comprehensive and validated QoL instruments tailored to penile cancer patients suggests that healthcare professionals may not fully capture the complexity of patients’ experiences. This can lead to gaps in care, particularly in areas such as sexual health, body image, and psychosocial support. Integrating more holistic and culturally sensitive assessments into routine care could improve patient-centered outcomes and support more individualized treatment planning.

From a research perspective, the results highlight the urgent need to develop and validate penile cancer-specific QoL instruments that encompass physical, emotional, social, and sexual dimensions. Future studies should also aim to include more diverse populations across different cultural and healthcare contexts to better understand regional variations in QoL. Additionally, longitudinal research is needed to explore how QoL evolves over time and how interventions can be tailored to different stages of the disease and recovery. Including the perspectives of partners and caregivers in future research could further enhance the understanding of psychosocial dynamics and support needs.

## Strengths and limitationss

The systematic review design has inherent limitations, as the included studies varied in terms of design, sample size, and methodologies, which should have been considered when drawing conclusions to ensure consistency.

Additionally, restricting the review to studies published in certain languages led to excluding some studies. Only peer-reviewed studies were selected to ensure the review was based on research of sufficiently high scientific standard, and the quality of the studies included in this review was evaluated as good. Multiple databases were utilized, and two authors independently conducted the review and performed the quality appraisal to limit reviewer and scoping bias. Due to institutional access constraints, Embase was not utilized in the literature search. It is known for its extensive biomedical coverage, and its omission may have resulted in the exclusion of some relevant studies. However, there is a substantial overlap between Embase and the other databases used—PubMed, CINAHL, PsycINFO, and the Cochrane Library. These databases were selected to ensure broad coverage of biomedical, nursing, psychological, and systematic review literature relevant to quality-of-life research in penile cancer.

The systematic review followed the methodology outlined by Bruce et al. (2018), supported by PRISMA guidelines and quality assessment tools, minimizing bias in study selection, data extraction, and synthesis, and ensuring rigorous quality assessment and reporting. The content analysis of this review was based on the descriptions of the content of the QoL instruments used in the reviewed articles. Notably, in some cases, the descriptions of the content, and therefore the material on which the content analysis of the QoL instruments was based, may have been insufficiently reported. In future studies, emphasis should be placed on the accuracy of reporting instruments and the specificity of their contents.

One limitation of this review is the lack of reported validity and reliability information for most of the QoL instruments used in the included studies. This gap restricts the extent to which the review could assess the psychometric soundness of the instruments, which was one of its primary aims. While such information is often available from original validation studies or official instrument documentation, these sources were not eligible for inclusion, unless they also assessed QoL in penile cancer patients.

## 5 Conclusions

Few studies have reported on the QoL of penile cancer patients, underscoring the necessity for increased focus on this aspect. The concentration of studies in Europe and the absence of research from other regions suggest a need for more geographically diverse studies to understand the global impact of penile cancer and its effect on patients' QoL. Furthermore, the findings of this study highlight the critical need for holistic instruments specifically designed to evaluate and enhance the QoL of penile cancer patients. Such instruments would ensure that these patients receive comprehensive care and support tailored to their unique needs. By developing and validating QoL tools for this specific patient group, healthcare providers can accurately identify and compare individual care needs and factors influencing their QoL. These tools would facilitate targeted interventions and enable healthcare providers to offer more personalized and effective care, addressing both the physical and psychological aspects of penile cancer.

## Funding sources

The research was partly funded by Finnish Cancer Foundation, Pirkanmaa Cancer Association and State Research Funding (VTR) for healthcare research.

## Data availability

The following materials are available upon request from the authors: data extracted from included studies, data used for all analyses, analytic code.

## CRediT authorship contribution statement

**Anu Soikkeli-Jalonen:** Writing – review & editing, Writing – original draft, Visualization, Supervision, Investigation, Formal analysis. **Suvi Vierelä:** Writing – review & editing, Writing – original draft, Visualization, Formal analysis, Data curation, Conceptualization. **Antti Kaipia:** Writing – review & editing, Supervision, Funding acquisition, Conceptualization. **Eeva Harju:** Writing – review & editing, Writing – original draft, Supervision, Investigation, Formal analysis, Conceptualization. **Elina Haavisto:** Writing – review & editing, Supervision, Project administration, Investigation, Funding acquisition, Formal analysis, Conceptualization.

## Declaration of competing interest

None.
